# Analysis of the photosynthetic apparatus in transgenic tobacco plants with altered endogenous cytokinin content: a proteomic study

**DOI:** 10.1186/1477-5956-9-33

**Published:** 2011-06-26

**Authors:** Anne Cortleven, Jean-Paul Noben, Roland Valcke

**Affiliations:** 1 Laboratory of Molecular and Physical Plant Physiology, Faculty of Sciences, Hasselt University, Diepenbeek, Belgium; 2 School of Life Sciences, Biomedical Research Institute and Transnational University Limburg, Hasselt University, Diepenbeek, Belgium

## Abstract

**Background:**

Cytokinin is a plant hormone that plays a crucial role in several processes of plant growth and development. In recent years, major breakthroughs have been achieved in the elucidation of the metabolism, the signal perception and transduction, as well as the biological functions of cytokinin. An important activity of cytokinin is the involvement in chloroplast development and function. Although this biological function has already been known for 50 years, the exact mechanisms remain elusive.

**Results:**

To elucidate the effects of altered endogenous cytokinin content on the structure and function of the chloroplasts, chloroplast subfractions (stroma and thylakoids) from transgenic P*ssu*-*ipt *and *35S:CKX1 *tobacco (*Nicotiana tabacum*) plants with, respectively, elevated and reduced endogenous cytokinin content were analysed using two different 2-DE approaches. Firstly, thykaloids were analysed by blue-native polyacrylamide gel electrophoresis followed by SDS-PAGE (BN/SDS-PAGE). Image analysis of the gel spot pattern thus obtained from thylakoids showed no substantial differences between wild-type and transgenic tobacco plants. Secondly, a quantitative DIGE analysis of CHAPS soluble proteins derived from chloroplast subfractions indicated significant gel spot abundance differences in the stroma fraction. Upon identification by MALDI-TOF/TOF mass spectrometry, these proteins could be assigned to the Calvin-Benson cycle and photoprotective mechanisms.

**Conclusion:**

Taken together, presented proteomic data reveal that the constitutively altered cytokinin status of transgenic plants does not result in any qualitative changes in either stroma proteins or protein complexes of thylakoid membranes of fully developed chloroplasts, while few but significant quantitative differences are observed in stroma proteins.

## 1. Background

Cytokinin is a class of plant hormones that affects multiple aspects of plant growth and development including cell division, vascular development, sink/source relations, apical dominance and leaf senescence [1,2]. Most steps of the cytokinin synthesis and breakdown [3], as well as its perception by membrane-located histidine kinases and transduction of the signal through a two-component signalling system, have been elucidated [4]. In addition, several activities of the hormone in regulating intrinsic developmental pathways and responses to environmental changes have been discovered [5]. However, an important activity of cytokinin, that was found soon after the discovery of cytokinin as a plant growth regulator, namely the involvement of cytokinin in chloroplast development, is poorly understood.

Several *in vitro *studies demonstrated that cytokinin can delay the yellowing of leaves (for review see [6]). Later, Stetler and Laetsch [7] reported a stimulation of chloroplast differentiation upon kinetin treatment. Parthier [8] also showed that exogenously applied cytokinin stimulates deetiolation, i.e. the transition of etioplasts into chloroplasts in detached leaves. Other studies showed that the biogenesis of chloroplasts is regulated by cytokinin providing components of the electron transport chain, structural and functional proteins and enzymes for the formation of the chloroplast stroma *in vivo*, and increases the life span of chloroplasts [1,9-12]. Cytokinin also affects pigment accumulation and the rate of photosynthesis [13-16]. Besides the effects on chloroplast structure and function itself, Okazaki *et al. *[17] revealed that cytokinin increases the chloroplast division rate. Chloroplasts themselves contain a broad set of cytokinins, including free bases, ribosides and N-glucosides [18]. Moreover, four cytokinin biosynthetic proteins (AtIPT1, AtIPT3, AtIPT5, and AtIPT8) are targeted to plastids [19].

Since the discovery of the effect of cytokinin on chloroplast development, most analyses have been performed on the effect of cytokinin on the transcript level [20-24]. Kusnetsov *et al. *[14] mentioned the stimulating effect of cytokinin on chloroplast proteins in *Lupinus luteus *cotyledons without notable effects on steady-state mRNA levels.

Expression profiling of cytokinin action in *Arabidopsis *or barley have been profoundly studied using endogenous altered cytokinin content or exogenous application of cytokinins. Hoth *et al. *[20] endogenously elevated the cytokinin content in *Arabidopsis *and revealed several targets of cytokinin responsive genes, especially genes involved in the cytokinin signal transduction pathway. Both Rashotte *et al. *[21] and Brenner *et al. *[22] applied exogenously cytokinins to *Arabidopsis *and found that the cytokinin-upregulated genes included, for example, transcription factors and cytokinin signalling proteins. In addition, Brenner *et al. *[22] showed that some genes involved in chloroplast development and function were upregulated. Further direct effects of the exogenous application of cytokinins on the activation of transcription of chloroplast genes in barley have been described by Zubo *et al. *[23,24]. They demonstrated that some genes (e.g. *PETD, ATPA, RRN16*) are highly responsive to cytokinin treatment, while other genes remain unaffected. Only a few proteomic studies have investigated the effect of cytokinin on chloroplasts. Lochmanova *et al. *[25] described the effects of cytokinin-induced photomorphogenesis in dark-grown *Arabidopsis *on the protein level. They reported that even a modest increase in the endogenous cytokinin level (< 2-fold increase) resulted in an upregulation of 37 proteins, mostly related to chloroplast biogenesis. More recently, Cerny *et al. *[26] described the early cytokinin response proteins and phosphoproteins in 7-day-old *Arabidopsis thaliana *plants. They not only confirmed the already known functions of cytokinin, but they also revealed that most differently regulated phosphoproteins are located in chloroplasts. Therefore, they suggested the presence of a not yet known signalling chain located in the chloroplast responsible for the cytokinin action.

In the past, effects of cytokinins were determined by applying cytokinins exogenously to the plants. This method is accompanied by many negative effects. High, non-physiological concentrations of the hormone are applied. Moreover, it is difficult to determine how much of the exogenously applied hormone is translocated in a biologically active form to a target tissue. A modulation of the cytokinin metabolism by a transgenic approach eliminates some of these problems. Several systems have been designed to overexpress the *IPT*-gene of *Agrobacterium tumefaciens*, encoding for isopentenyltransferase, which is a key enzyme of the cytokinin biosynthetic pathway, to obtain elevated endogenous cytokinin content (heat-shock promoter [27]; tetracycline inducible promoter [28]). The use of heat-shock promoters induces additional changes, since a heat-shock has to be applied for the induction of the promoter. Profound analysis of the transgenic plants with a tetracycline inducible promoter showed that no elevation of cytokinin could be found in the leaves [29]. In this study, two transgenic tobacco plant models (P*ssu-ipt *and *35S:CKX1*) were studied. Transgenic (P*ssu-ipt*) [30] tobacco plants express *IPT *under control of a light-inducible promoter of the RuBisCo enzyme of *Pisum sativum*, and *35S:CKX1 *transgenic tobacco plants express a gene for cytokinin oxidase/dehydrogenase from *A. thaliana *under control of a constitutive CaMV 35S promoter and show diminished cytokinin content [31]. A correlation between the expression of the transgene and the altered endogenous cytokinin content has been proven in [32]. To our knowledge, no transcription/proteomic data reports exist on transgenic tobacco plants with diminished endogenous cytokinin content.

Functional analysis, using chlorophyll *a *fluorescence kinetics, and pigment analysis have already shown that cytokinin affects the photosynthetic apparatus [[31], unpublished observations]. While decreased endogenous cytokinin content diminished the chlorophyll biosynthesis without a significant effect on the photosynthetic activity [31], increased endogenous cytokinin content elevated the chlorophyll content and caused functional differences in the activity of the different parts of the photosynthetic electron transport chain [[33], unpublished observations]. Moreover, the ultrastucture of chloroplasts is dramatically changed in both transgenic tobacco plants [9,31].

To investigate if these functional and structural differences are a consequence of alterations in the chloroplast protein composition, two different proteomic approaches are used: BN/SDS-PAGE and IEF/SDS-PAGE in combination with the DIGE technology. IEF/SDS-PAGE has been proven to resolve large numbers of soluble and peripheral membrane proteins [34,35]. However, this technique is not very useful for the separation of highly hydrophobic membrane proteins present in the thylakoid membranes of chloroplasts, such as photosystem I, photosystem II, cytochrome *b*_*6*_*f*, and ATP-synthase [36]. These membrane proteins are poorly soluble in the commonly used IEF compatible detergents (such as CHAPS) and tend to precipitate near their isoelectric point. As an alternative, BN/SDS-PAGE is used. This technique is a better method to resolve highly hydrophobic integral membrane proteins from thylakoid membranes [37]. Here it is used to resolve digitonin-solubilized hydrophobic thylakoid proteins and complexes thereof in the first dimension, allowing for a qualitative study of the protein complex polypeptide composition in the second dimension in wild-type and transgenic plants. Finally, DIGE is used to quantitatively analyse the stromal proteome in the same experimental settings. In addition to this proteome analysis, we also investigated the transcription levels of genes encoding proteins that were found to be differently expressed in stroma in response to altered endogenous cytokinin content.

## 2. Results

The studied transgenic *35S:CKX1 *and P*ssu-ipt *tobacco plants show a severely retarded development and differences in growth pattern compared to their corresponding wild-types (respectively Samsun NN and SR1). These morphological and developmental differences as a consequence of the expression of the transgene have already been described by Werner et al. [31,38,39] and by Synková et al. [33].

### 2.1. BN/SDS-PAGE of thylakoids

Isolated thylakoids from plants with altered endogenous cytokinin content were first submitted to BN-PAGE. For this purpose, thylakoid membranes were treated with digitonin, which is known to be a suitable detergent for the solubilisation and stabilisation of supercomplexes [37,40].

Ten different protein complexes were identified using MALDI-TOF/TOF in combination with previously published data [40]. The upper five bands can be assigned to supercomplexes with a high molecular mass. They contain PSI/PSII and associated LHC together with ATP-synthase. Band F corresponds to a supercomplex consisting of PSI/LHCI and PSII (dimeric core). RuBisCO is the main component of band G. The presence of RuBisCO in a thylakoid fraction has already been shown by Aro *et al. *[41] and Timperio *et al. *[42]. Band H can be assigned to the PSII/ATP-synthase complex and band I to cytochrome *b*_*6*_*f*. Band J represents the trimeric light-harvesting complex III.

**Figure 1:**
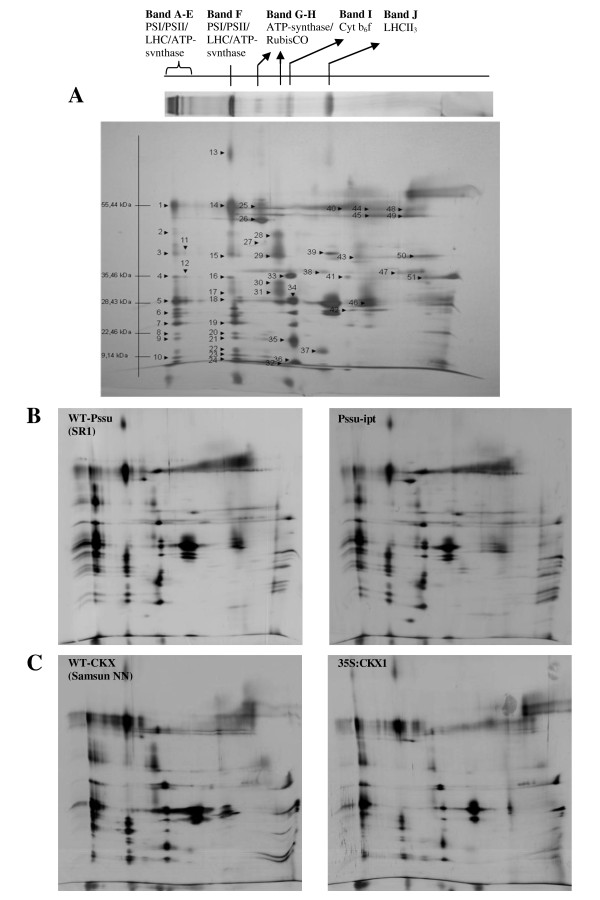
**Effect of endogenous altered cytokinin content on the protein subunit composition of the thylakoid membranes**. Chloroplast proteins were solubilized in 1% digitonin and separated in a first native gel electrophoresis (BN-PAGE). Protein subunits were released from protein complexes and were resolved by SDS-PAGE in a second dimension (SDS-PAGE). (A) Average 2D-BN/SDS-PAGE map of *Nicotiana tabacum *thylakoid membranes. The different protein complexes are indicated above the gel. Identified proteins are indicated with a number and an overview of identified proteins can be found in table 1 and in Additional file 1, table S1. (B-C) 2D-BN/SDS-PAGE map of P*ssu-ipt *(B) and *35S:CKX1 *(C) and corresponding wild-type (WT). (PSI: photosystem I; PSII: photosystem II; LHC: light-harvesting complex; Rubisco: ribulose-bisphosphate carboxylase; cyt *b*_*6*_*f*: cytochrome *b*_*6*_*f*; LHCII_3_: trimeric light-harvesting complex).

Blue-native separated protein complexes were directly transferred and analysed in a second dimension gel using denaturing SDS-PAGE. Under these conditions, protein complexes dissociate into their subunits, and the protein subunits of the different complexes are separated according to their molecular mass. Figure 1A gives a representative spot map of wild-type P*ssu-ipt *tobacco thylakoids. An overview of identified spots is given in Table 1 and supplemental data about the identified proteins can be found in Additional file 1, table S1. It should be noted that not all proteins can be identified by this experimental approach. Some proteins are deficient in the basic amino acids arginine and lysine and, therefore, are poorly proteolysed by trypsin. Alternatively, hydrophobic tryptic fragments might be lost during peptide extraction prior to mass spectrometry.

**Table 1:** Proteins identified by MALDI-TOF/TOF from the second dimension BN/SDS-PAGE.

Spot number	Protein Name	SwissProt Accession number	MW	pI
1	ATP synthase subunit alpha, chloroplastic [*Nicotiana tabacum*]	P00823	55477,1	5,14

2	Photosystem II CP47 chlorophyll apoprotein [*Cucumis sativus*]	Q2QD63	55982,37	6,28

3	Photosystem II CP43 chlorophyll apoprotein [*Ranunculus macranthus*]	Q4FFN5	52017,79	6,68

4	Oxygen-evolving enhancer protein 1, [chloroplastic *Nicotiana tabacum*]	Q40459	35377,09	5,89

5	Chlorophyll a-b binding protein 40, chloroplastic [*Nicotiana tabacum*]	P27495	28450,22	5,48

6	Chlorophyll a-b binding protein 13, chloroplastic [*Solanum lycopersicum*]	P27489	28661,44	5,09

7	Chlorophyll a-b binding protein 6A, chloroplastic [*Solanum lycopersicum*]	P12360	26785,52	5,82

8	Photosystem I reaction center subunit II, chloroplastic [*Nicotiana sylvestris*]	P29302	22466,7	9,78

9	Photosystem I reaction center subunit II, chloroplastic [*Nicotiana sylvestris*]	P29302	22466,7	9,78

10	Photosystem I reaction center subunit III, chloroplastic [*Spinacia oleracea*]	P12355	25567,52	9,4

11	Photosystem II CP43 chlorophyll apoprotein [*Aethionema grandiflora*]	A4QJJ5	52042,77	6,71

12	Oxygen-evolving enhancer protein 1, chloroplastic [*Nicotiana tabacum*]	Q40459	35377,09	5,89

13	Ribulose bisphosphate carboxylase large chain (Fragment) [*Nelumbo lutea*]	Q05800	44231,36	6,4

14	Photosystem I P700 chlorophyll a apoprotein A1 [*Nicotiana tomentosiformis*]	Q33C36	83206,56	6,67

15	Photosystem II CP43 chlorophyll apoprotein [*Ranunculus macranthus*]	Q4FFN5	52017,79	6,68

16	Oxygen-evolving enhancer protein 1, chloroplastic [*Nicotiana tabacum*]	Q40459	35377,09	5,89

17	Photosystem II D2 protein [*Dioscorea elephantipes*]	A6MMK2	39765,89	5,34

18	Chlorophyll a-b binding protein 40, chloroplastic [*Nicotiana tabacum*]	P27495	28450,22	5,48

19	Chlorophyll a-b binding protein 6A, chloroplastic [*Solanum lycopersicum*]	P12360	26785,52	5,82

20	Photosystem I reaction center subunit II, chloroplastic [*Nicotiana sylvestris*]	P29302	22466,7	9,78

21	Photosystem I reaction center subunit II, chloroplastic [*Nicotiana sylvestris*]	P29302	22466,7	9,78

22	Photosystem I reaction center subunit IV B, chloroplastic [*Nicotiana sylvestris*]	Q41229	15214,74	9,74

23	Photosystem I reaction center subunit III, chloroplastic [*Flaveria trinervia*]	P46486	25366,48	9,35

24	Cytochrome b559 subunit alpha [*Arabidopsis thaliana*]	P56779	9380,7	4,83

25	ATP synthase subunit alpha, chloroplastic [*Nicotiana tabacum*]	P00823	55477,1	5,14

26	Ribulose bisphosphate carboxylase large chain [*Nicotiana sylvestris*]	Q3C1J4	53377,98	6,41

27	Ribulose bisphosphate carboxylase large chain (Fragment) [*Adoxa moschatellina*]	P28378	52159,14	6,13

28	Photosystem II CP47 chlorophyll apoprotein [*Barbarea verna*]	A4QKD1	56204,47	6,4

30	Photosystem Q(B) protein [*Leptosira terrestri*]	A6YGB8	38353,08	5,52

31	Photosystem Q(B) protein [*Leptosira terrestris*]	A6YGB8	38353,08	5,52

32	Cytochrome b559 subunit alpha [*Arabidopsis thaliana*]	P56779	9380,7	4,83

33	Apocytochrome f [*Nicotiana tabacum*]	P06449	35337,77	9,12

35	Cytochrome b6-f complex iron-sulfur subunit 2, chloroplastic [*Nicotiana tabacum*]	Q02585	24491,28	8,15

36	Cytochrome b6-f complex subunit 4 [*Agrostis stolonifera*]	A1EA39	17534,51	6,56

37	ATP synthase subunit b, chloroplastic [*Nicotiana tabacum*]	yP06290	20917,96	8,76

38	Ferredoxin--NADP reductase, leaf-type isozyme, chloroplastic [*Nicotiana tabacum*]	O04977	40704,59	8,37

39	Fructose-bisphosphate aldolase, chloroplastic [*Spinacia oleracea*]	P16096	42726,8	6,85

40	Photosystem II 22 kDa protein, chloroplastic [*Nicotiana tabacum*]	Q9SMB4	29068,73	6,04

41	Apocytochrome f [*Nicotiana tabacum*]	P06449	35337,77	9,12

42	Photosystem II 22 kDa protein, chloroplastic [*Nicotiana tabacum*]	Q9SMB4	29068,73	6,04

43	Photosystem II CP43 chlorophyll apoprotein [*Aethionema grandiflora*]	A4QJJ5	52042,77	6,71

44	ATP synthase subunit beta, chloroplastic [*Nicotiana plumbaginifolia*]	P69370	53548,81	5,09

45	ATP synthase subunit alpha, chloroplastic [*Nicotiana tabacum*]	P00823	55477,1	5,14

46	Chlorophyll a-b binding protein CP26, chloroplastic [*Arabidopsis thaliana*]	Q9XF89	30194,7	6

47	Ferredoxin--NADP reductase, leaf-type isozyme, chloroplastic [*Nicotiana tabacum*]	O04977	40704,59	8,37

48	ATP synthase subunit alpha, chloroplastic [*Nicotiana tabacum*]	P00823	55477,1	5,14

49	ATP synthase subunit alpha, chloroplastic [*Nicotiana tabacum*]	P00823	55477,1	5,14

50	ATP synthase gamma chain, chloroplastic [*Nicotiana tabacum*]	P29790	41705,96	8,16

51	ATP synthase subunit beta, chloroplastic [*Nicotiana tabacum*]	P00826	53577,78	5

52	Oxygen-evolving enhancer protein 1, chloroplastic [*Nicotiana tabacum*]	Q40459	35377,09	5,89

Additional information about the identified proteins can be found in Additional file 1, table S1.

The spot maps obtained from the wild-type tobacco and corresponding transgenic thylakoids were superimposable with no discernable qualitative difference upon image analysis (Figure 1B). Quantitative differences were not taken into consideration.

### 2.2. IEF/SDS-PAGE of chloroplast fractions (DIGE)

The stroma fraction from tobacco chloroplasts was solubilised in a chaotropic CHAPS sample buffer and subjected to 2D-DIGE, as described in section 5.5. Thylakoid membrane proteins are not easily solubilised with used DIGE sample buffer and can cause problems in IEF, as has already been mentioned above. With this constraint in mind, we performed DIGE analysis of both the thylakoid (data not shown) and the stroma fraction.

**Figure 2:**
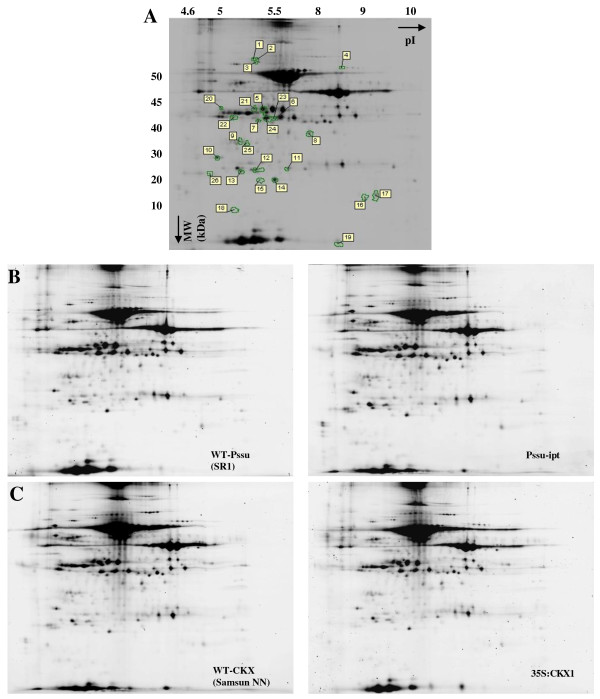
**Effect of endogenous altered cytokinin content on the proteome of the stroma fraction**. Proteins were separated in two dimensions: according to isoelectric point (pI) and molecular weight (MW). (A) Average two-dimensional gel electrophoresis proteome map. Differently regulated protein spots are indicated and identified by MALDI-TOF/TOF. An overview of identified proteins can be found in table 2 and in Additional file 2, table S2. (B-C) Proteome map of the stroma fraction of P*ssu-ipt *(B) and *35S:CKX1 *(C) and corresponding wild-type (WT).

Figure 2A shows a representative gel image of the stroma fraction containing about 1,500 spots. Comparing P*ssu-ipt *and *35S:CKX1 *with corresponding wild types (Figure 2B-C) showed that 19 spots (Figure 2A, spots labelled 1 to 19) and 9 spots, respectively, (Figure 2A, spots labelled 20 to 26, 6 and 17) were differentially abundant (p < 0.05; spot abundance ratio ≥ ± 1.2). Overall mass spectrometric identification rate was unexpectedly low and 10 spots in total could be identified. These included proteins related to the Calvin-Benson cycle (spots 6, 23 and 24), proteins involved in proteolytic processes in chloroplasts (spot 3), in the final step of the electron transport (spot 8), in protection of chloroplasts to oxidative damage (spot 14), and in the oxygen evolving complex (spot 13). An overview of the differently expressed protein spots and identification is given in Table 2. Additional information about the identified proteins can be found in Additional file 2, table S2.

**Table 2:** Summary of proteins different in spot abundance ratio between wild-type and transgenic tobacco plants in stroma fraction.

*Stroma fraction Pssu-ipt versus wild-type (SR1)*					
**Spot number**	**Protein name + SwissProt accession number**	**MW**	**pI**	**Fold change**	**p-value**

1	Not identified	-	-	1.76	0.012

2	Not identified	-	-	1.71	0.047

3	ATP-dependent Clp protease ATP-binding subunit clpA homolog CD4B, chloroplastic [*Solanum lycopersicum*] P31542	102462.8	5.86	1.98	0.039

4	Not identified	-	-	-1.44	0.023

5	Not identified	-	-	4.47	0.025

6	Probable fructose-bisphosphate aldolase 1, chloroplastic [*Arabidopsis thaliana*] Q9SJU4	43074.98	6.18	2	0.0114

7	Not identified	-	-	1.91	0.0114

8	Ferredoxin--NADP reductase, leaf-type isozyme, chloroplastic [*Nicotiana tabacum*] O04977	40704.59	8.37	1.8	0.0134

9	Not identified	-	-	2.18	0.046

10	Not identified	-	-	1.99	0.0114

11	Not identified	-	-	1.9	0.039

12	Not identified	-	-	2.23	0.043

13	Oxygen-evolving enhancer protein 2-1, chloroplastic [*Nicotiana tabacum*] Q7DM39	28805.44	6.84	3.02	9.40E-03

14	Superoxide dismutase [Fe], chloroplastic (Fragment) [*Nicotiana plumbaginifolia*] P22302	23027.52	5.53	4.84	6.70E-03

15	Not identified	-	-	2.97	6.7E-03

16	Not identified	-	-	2.37	0.0146

17	Not identified	-	-	1.96	0.0146

18	Not identified	-	-	1.48	0.0146

19	Not identified	-	-	-1.57	6.7E-03

					
***Stroma fraction 35S:CKX1 versus wild-type *(Samsun NN)**					

**Spot number**	**Protein name + SwissProt accession number**	**MW**	**pI**	**Fold change**	**p-value**

6	Probable fructose-bisphosphate aldolase 1, chloroplastic [*Arabidopsis thaliana*] Q9SJU4	43074.98	6.18	1.42	5.46E-03

17	Not identified	-	-	-1.46	5.46E-03

20	Not identified	-	-	-1.45	5.46E-03

21	Not identified	-	-	1.45	0.0103

22	Not identified	-	-	1.24	9.43E-03

23	Probable fructose-bisphosphate aldolase 1, chloroplastic [*Arabidopsis thaliana*] Q9SJU4	43074.98	6.18	1.36	5.46E-03

24	Probable fructose-bisphosphate aldolase 1, chloroplastic [*Arabidopsis thaliana*] Q9SJU4	43074.98	6.18	1.39	5.46E-03

25	Not identified	-	-	1.3	9.43E-03

26	Not identified	-	-	-1.66	9.43E-03

Proteins were identified by MALDI-TOF/TOF. The fold change indicates the difference in expression relative to wild-type plants. Additional information about the identified proteins can be found in Additional file 2, table S2.

### 2.3. Quantitative real-time PCR

Proteome analysis of the stroma fraction showed that the abundance of several proteins was affected by cytokinin. Transcript level of their encoding genes (*NtFNR*, *NtSODB*, *NtPSBO*, *NtPSBP*, and *NtPSBQ*) were quantified by RT-PCR using the appropriate reference genes (*NtRBCS, NtTUB and NtACT9) *[32].

**Figure 3:**
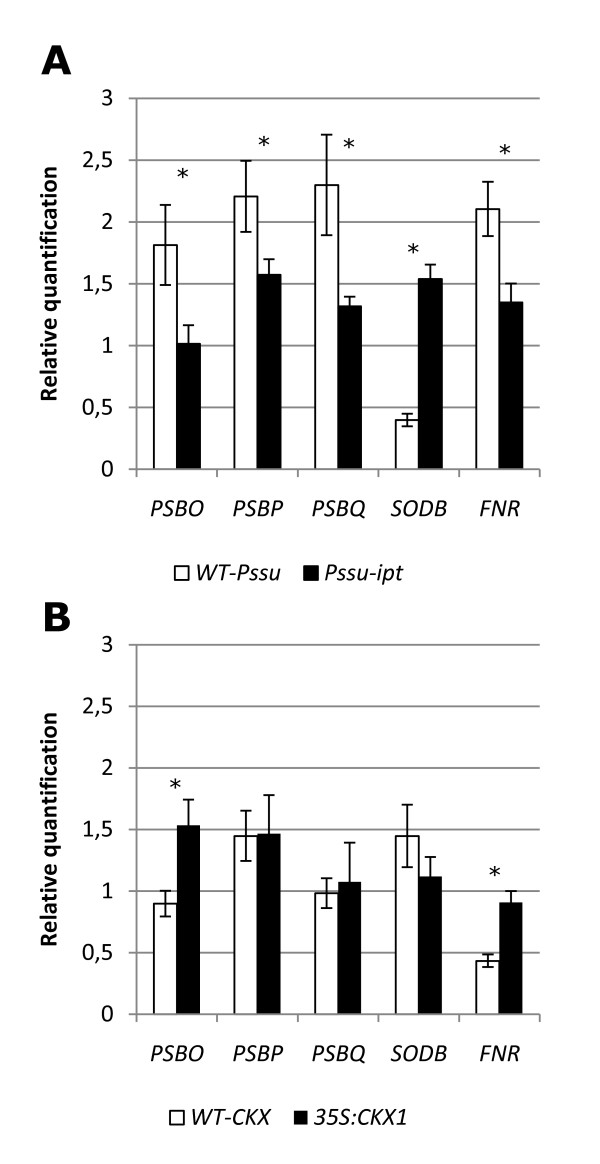
**Effect of endogenous altered cytokinin content on transcript**. Transcription levels of genes encoding differently expressed proteins in transgenic tobacco plants (*NtFNR, NtPSBP, NtSODB*) and genes encoding subunits of the OEC (*NtPSBO *and *NtPSBQ*) for (A) transgenic P*ssu-ipt *tobacco plants and (B) *35S:CKX1 *tobacco plants and corresponding wild-types. All values are presented with SE. Statistical significant differences (p < 0.05) are indicated (*).

* NtSODB *is highly expressed in P*ssu-ipt *tobacco plants, while for *NtFNR*, *NtPSBO*, *NtPSBP*, and *NtPSBQ*, the opposite observation is made (Figure 3A). Within the *35S:CKX1 *tobacco plants the expression levels of only *NtFNR *and *NtPSBO *are elevated, (Figure 3B) in comparison with wild-type plants. For fructose-1,6-bisphosphate aldolase, which is found to be affected by cytokinin in this study, quantification of the associated transcript is not possible since sequence details are not yet publicly available for this *Nicotiana tabacum *enzyme.

## 3. Discussion

### 3.1. BN/SDS-PAGE and IEF/SDS-PAGE complement each other to study the effects of cytokinins on the proteome of chloroplasts

Although BN/SDS-PAGE was restricted to the thylakoid membrane fraction, we found the presence of some stroma proteins (RuBisCO, plastidic aldolase and ferredoxin-NADP reductase). This could be due to contamination, although it is possible that these proteins associate with the thylakoid membrane to function more efficiently. This kind of association has already been demonstrated in *Anacystis nidulans*, where several enzymes involved in the Calvin-Benson cycle are closely associated with the thylakoid membranes to get better access to ATP and NADPH, produced during the electron transfer [43]. The presence of RuBisCO in BN-gels has also been shown by Aro *et al. *[41] and Timperio *et al. *[42].

Our results indicate that there is no qualitative difference in the oligomeric assembly state of protein complexes and subunit composition between transgenic and wild-type plants, as judged from proteome maps generated by BN/SDS-PAGE. However, changes observed in photosynthetic activity in response to altered endogenous cytokinin content [33,39] may still be related to a quantitative difference in subunit composition of the membrane protein complexes.

The easily soluble stroma fraction was subjected to IEF/SDS-PAGE to obtain information on the quantitative changes induced by elevated/reduced endogenous cytokinin content in the chloroplast. IEF/SDS-PAGE of the fluorescent labelled stroma fraction showed that there were only a few quantitative differences in protein composition between the wild-type and transgenic plants. These differences are related to proteolytic processes, electron transport, protection against oxidative damage, and the oxygen evolving complex.

Both chloroplastic superoxide dismutase (Fe) (spot 14) and ferredoxin-NADP+ reductase (spot 8) have a higher protein abundance in P*ssu-ipt *transgenic tobacco. Both proteins can be correlated to protection against photo-oxidative damage. Superoxide is formed at any location in which an electron transport chain and O_2 _are present, such as in photosystem I in chloroplasts (Mehler-reaction). It is believed that the major task of chloroplastic SOD in plants is to remove superoxide that is produced by electron leakage to oxygen at the reducing site of photosystem I. FeSOD is localised in the chloroplasts and involved in the protection of both the plasmamembrane and PSII against oxidative damage [44]. This observation corresponds to the study of [45], who demonstrated that cytokinin induces the activity of antioxidant enzymes during ontogeny. The increased amount of FNR, which catalyses the electron transfer from ferredoxin to NADP^+^, can also be correlated to photo-oxidative protection. A recent study demonstrated that tobacco plants overexpressing ferredoxin-NADP^+^reductase showed augmented tolerance to photo-oxidative damage and redox-cycling oxidants [46]. Cerny *et al. *[26] also found an increased level of FNR in the early cytokinin-response proteins.

The chloroplast homologue of ClpA (Caseinolytic protease - designated ClpC), which has a higher protein level in P*ssu-ipt *tobacco plants, is encoded in the nucleus [47] and imported posttranslationally into the stroma. It is an ATP-dependent unfoldase involved in the degradation of mistargeted proteins in the stroma and the degradation of chloroplast protein complexes with an unbalanced stoichiometry and damaged proteins by feeding the unfolded proteins to the ClpP proteolytic complex. It is known that Clp proteases also play an important role in the development of chloroplasts in *Arabidopsis thaliana *[48-50]. This suggests that there could be a correlation between the higher protein level of ClpA and chloroplast development in P*ssu-ipt *tobacco plants. These plants have chloroplasts with a specific ultrastructure: they contain more grana stackings and increased starch grains, as described by Synková *et al. *[9]. Moreover, Lochmanova *et al. *[25]. found an induction of a Clp protease.

Fructose bisphosphate aldolase (spots 6, 23 and 24), which catalyses the conversion of glyceraldehyde 3-phosphate with dihydroxyacetone to fructose 1,6-bisphosphate, has an increased protein level in both P*ssu-ipt *and in *35S:CKX1 *tobacco plants. The higher protein level in *35S:CKX1 *is in correspondence with the higher enzyme activity, as demonstrated by Werner *et al. *[31]. They correlated the higher activity of aldolase with a compensation mechanism to a lowered sugar status as a consequence of the altered sink-to-source relation. The increased level in P*ssu-ipt *tobacco plants is in correspondence with Cerny *et al. *[26], who also found an upregulation of fructose bisphosphate aldolase in *Arabidopsis thaliana *after induction with cytokinin.

Remarkably, there was only a small amount of differentially expressed proteins in our study. Although this is not completely unexpected, since we used only the purified stroma fraction for analysis, while other studies often use total protein extracts [25,26]. Moreover, these studies used a different system to investigate the effect of cytokinin: exogenous application of cytokinin to 7-day-old *Arabidopsis *seedlings [26] or a dexamethasone-inducible system [25], which makes a direct comparison to our study difficult.

Comparison of the proteome results obtained by the analysis of transgenic tobacco plants with elevated/diminished endogenous cytokinin content compared to their corresponding wild-types revealed that only one common protein, fructose-bisphosphate aldolase 1, was similarly influenced by the altered cytokinin status. This can be explained by the different genetic background of both transgenic plants and by the different mode of action of cytokinin.

### 3.2. Transcription level of photosynthetic genes and comparison with proteome data

We evaluated the transcript levels of genes encoding proteins that were differently expressed in the studied transgenic tobacco plants. A straightforward correlation between the transcript and protein level is however not possible. This has already been indicated by Gygi *et al. *[51]. Moreover, in our analysis, material of different leaves was used for proteome and transcript analyses. Nevertheless, quantitative real-time PCR showed that the transcript levels of the investigated genes are differently expressed in the transgenic plants.

The decreased transcript levels of the investigated genes in P*ssu-ipt *tobacco plants and opposite effects on mRNA levels (for *PSBO *and *FNR*) in cytokinin deficient plants can be explained by the effect of cytokinins on the complex signalling process that controls the expression of most nuclear-encoded and some plastid-encoded photosynthesis genes. The redox state of the electron transport chain, tetrapyrrole intermediates and reactive oxygen species provide a positive and negative feedback loop to regulate the expression of the photosynthesis genes and these processes can be influenced by cytokinins [16,33,52].

The higher expression in P*ssu-ipt *transgenic tobacco plants is possibly a consequence of the effects of cytokinin on electron transfer (reduced activity at PSI results in the production of superoxide) to induce oxidative stress. A complex control network is activated to reduce the effects of oxidative stress and the transcriptional regulation of *NtSODB *is possibly part of this network.

These results clearly indicate that the transcriptional regulation of photosynthesis genes and the involvement of cytokinin in this regulation network is rather complex and needs further elucidation.

## 4. Conclusion

In this study, we have evaluated the role of cytokinin in the structure and function of the photosynthetic apparatus. Although cytokinin is known to have distinct effects on chloroplast ultrastructure and function, only a few differences were found on protein level. The altered endogenous cytokinin content does not structurally change the subunit composition of the supercomplexes, and IEF/SDS-PAGE showed that cytokinins induce some significant changes in the proteome of the stroma fraction, which could be correlated to protection against oxidative stress.

## 5. Materials and methods

### 5.1. Plant material and growth conditions

All plants were grown in a greenhouse during spring time (temperature 24°C/18°C, average humidity 60%). Additional illumination was provided 16 h a day with AgroSon T (400W) and HTQ (400W) lamps (photon flux density of 200 μmol quanta (m^-2^s^-1^)). Two different types of transgenic tobacco plants with altered cytokinin metabolism and the corresponding wild types were used.

1. Transgenic tobacco plants (*Nicotiana tabacum *L. cv. Petit Havana SR1) containing the *IPT*-gene under control of the *Pisum sativum *ribulose-1,5-biphosphate carboxylase small subunit promoter sequence (P*ssu-ipt*) were obtained using the *Agrobacterium tumefaciens *system, as described by Beinsberger *et al. *[30]. After transformation, the seeds were sown on Murashige and Skoog medium with kanamycin (100 mg/ml). Corresponding wild-type plants (SR1) were cultivated under the same conditions without kanamycin. Only kanamycin resistant transgenic seedlings (2-3 weeks old) were transferred to potting soil (Universal potting soil, Agrofino, Agrofino Products N.V.) and further cultivated under the same conditions as wild-type plants. Soil was supplemented with half-strength Hoagland solution.

2. Tobacco plants (*Nicotiana tabacum *L. var. Samsun NN) (*35S:AtCKX1*), overexpressing a gene for cytokinin oxidase/dehydrogenase from *Arabidopsis thaliana *under control of a constitutive CaMV 35S promoter [38], were first cultivated *in vitro *on Murashige and Skoog medium with hygromycin (15 mg/l). Corresponding wild-type plants (Samsun) were cultivated under the same conditions without hygromycin. The hygromycin resistant seedlings and wild-type plants were transferred to potting soil under the same conditions as described for the P*ssu-ipt *plants.

To homogenize the experiments, plants of the same height were used: 7-week-old wild-type plants (SR1: 32.7 ± 4.1 cm; n = 15 and Samsun: 7.5 ± 1.4 cm; n = 15), 18-week-old P*ssu-ipt *plants (35 ± 2 cm; n = 15) and 14-week-old *CKX *tobacco plants (7.5 ± 1.1 cm; n = 15).

### 5.2. Isolation of intact chloroplasts and chloroplast fractions

In each out of four independent experiments, intact chloroplasts were isolated from the upper four leaves larger than 5 cm of six plants of each of the two transgenic and the two wild-type lines, following a modified method described in Bartlett [53]. Leaves (50 g) were harvested at the same time in the morning and homogenized in 200 ml of ice-cold grinding buffer (2.0 mM NaEDTA; 1.0 mM MgCl_2_; 1.0 mM MnCl_2_; 50.0 mM Hepes/KOH, pH 7.5; 0.33 M sorbitol; 5.0 mM sodiumascorbate) using a Braun MX-32 mixer. The suspension was filtered through four layers of miracloth (pore size: 22-25 μm) and chloroplasts were sedimented by centrifugation (1400 g, 5 min). The pellet was resuspended in grinding buffer (5 ml/50 g) and 2 ml of this suspension was loaded on a continuous 10-80% Percoll gradient (3% PEG 6000; 1% Ficoll; 1% BSA) and centrifuged for 20 min at 8000 g. Two green bands were visible after centrifugation. The lower band containing the intact chloroplasts was collected, washed with 5-10 volumes of grinding buffer and centrifuged for 10 min. The intact chloroplasts were used for the isolation of thylakoid for BN and for chloroplast subfraction isolation. For BN, the intact chloroplasts were resuspended in TMK-buffer (10 mM Tris pH 6.8; 10 mM MnCl_2_; 20 mM KCl) and centrifuged for 10 min at 2200 g. The pellet was solved in TMK buffer (1 mg chlorophyll/ml) and stored at -70°C.

To obtain the chloroplast subfractions, the intact chloroplasts were resuspended in 3 ml resuspension buffer (25 mM HEPES pH 7.5, 10 mM EDTA). The resuspended chloroplasts were homogenized using a glass homogenizer with a Teflon pestle, put on top of a discontinuous sucrose gradient (1.9 M, 1.30 M and 1.14 M sucrose in 25 mM HEPES pH 7.5) and centrifuged for 90 min at 100,000 g. After centrifugation, different chloroplast fractions can be distinguished: the stroma fraction (top of the gradient), the envelope fraction and the thylakoid fraction (dark green bands). The stroma fraction was frozen at -70°C until protein extraction.

### 5.3. Protein extraction of chloroplast subfraction

Proteins from the thylakoid fraction were extracted with 9 ml chloroform/methanol (7:2). The resulting mixture was put on ice for 20 min before centrifugation (20 min at 12,000 g). The pellet was dried under N2 and solubilized in DIGE sample buffer (7 M urea, 2 M thiourea, 4% CHAPS, 30 mM Tris, pH 8.8).

Proteins from the stroma were concentrated using TCA (trichloro-acetic acid) precipitation. An equal amount of ice-cold 20% TCA (w/v) was added to the stroma fraction and incubated for 1 h at 4°C. After incubation, the suspension was centrifuged for 10 min at 3000 g. The pellet was washed with methanol-diethylether (1:1) and centrifuged under the same conditions. Afterwards, the pellet was washed with 100% diethylether and centrifuged again under the same conditions. The pellet of the last centrifugation was dried under N_2 _and solubilized in DIGE sample buffer (7 M urea, 2 M thio-urea, 4% CHAPS). The protein concentration of these samples was determined by using the 2D-Quant-Kit (GE Healthcare/Amersham Biosciences, Freiburg, Germany).

### 5.4. BN/SDS-PAGE

#### BN-PAGE/SDS-PAGE

BN-PAGE was performed according to Reisinger *et al. *[54]. Before solubilization, the enriched thylakoid suspension (in TMK buffer) was centrifuged at 400 g for 3 min. The pelleted thylakoids (50 μl) were then solubilized with 2.5% (w/v) digitonin for 10 min at 4°C. After solubilization, the samples were centrifuged for 10 min at 13,000 g. One μl loading buffer (5% (w/v) Serva Blue G250, 750 mM ε-aminocaproic acid) was added to the supernatans and the mixture was loaded on a 5-15% acrylamide gradient gel (4% acrylamide stacking gel). Electrophoresis was performed in a Protean II electrophoresis system (Bio-Rad, USA) at 4°C, applying a constant voltage of 250 V overnight. For SDS-PAGE, lanes of the BN-PAGE were first incubated in solubilization buffer (2% (w/v) SDS, 66 mM Na_2_CO_3_, 2% (w/v) β-mercapto-ethanol, 10% (w/v) glycerol, 0.5 M Tris/HCl, pH 6.8) for 30 min at room temperature, subsequently put on a second dimension gel (10% acrylamide; stacking gel: 4% acrylamide) and overlaid with agarose solution (25 mM Tris/HCl, pH 8.8, 192 mM glycine, 0.1% SDS, 0.5% (w/v) agarose, 0.0002% bromophenolblue). Electrophoresis (1 h at 2 W/gel, followed by 1 h at 4 W/gel and, finally, 4 h at 17 W/gel) was carried out in the EttanDALT*six *system (GE Healthcare).

#### Staining and data analysis

The gels were fixed in fixation solution (45% (v/v) methanol, 5% (v/v) acetic acid) overnight. The BN-gels (first dimension) were stained with Coomassie dye (50% (v/v) methanol, 7% (v/v) acetic acid, 0.025% (w/v) Coomassie Brilliant Blue G250) for 4 h and destained overnight in destaining solution (50% (v/v) methanol, 7% (v/v) acetic acid); after fixation, the second dimension gels were washed three times with 50% (v/v) ethanol for 20 min, incubated for 1 min in 0.02% (w/v) Na_2_SO_4 _and rinsed three times with water. Thereafter, the gels were incubated for 20 min in 0.2% (w/v) silver nitrate, rinsed three times with MilliQ water and incubated in 0.04% formaldehyde and 6% (w/v) sodium carbonate until the spots were visible. The staining reaction was stopped using 5% (v/v) acetic acid. After staining, the gels (in duplicate) of four biologically independent chloroplast extractions were scanned and analysed with Image Master Platina (Amersham Bioscience).

#### Proteolysis and peptide extraction

The protein spots from the second dimension (BN/SDS-PAGE) were detected using Image Master 5.0 (GE Healthcare), selected for picking by a gel cutting station (ProxCision, PerkinElmer, USA) and collected in a microtiter plate (ThermoFast semi skirted, Abgene, UK). The gel plugs were subjected to two wash steps: water (5% (v/v) CH_3_CN) and acetonitrile (5% (v/v) H_2_O) on a liquid handling robot (MultiProbeII HT, PerkinElmer). The dehydrated gel particles were rehydrated in 5 μl of digest buffer containing 83 ng trypsin (MS Gold; Promega, USA), 50 mM NH_4_HCO_3 _and 5% (v/v) CH_3_CN for 16 min at 4°C. After the addition of 15 μl of a buffer containing 50 mM NH_4_HCO_3 _and 5% (v/v) CH_3_CN, proteins were digested at 38°C for 3h. The resulting peptides were desalted and concentrated with microcolumn solid phase tips (PerfectPuretM C18 tip, 200 nl bed volume; Eppendorf, Germany) and eluted directly onto a MALDI target plate (OptiMALDI; Applied Biosystems, USA) with 1.0 μl of 50% CH_3_CN, 0.1% CF_3_COOH solution saturated with cyano-4-hydroxycinnamic acid and spiked with 25 fmol/μl Glu1-fibrinopeptide B (Sigma-Aldrich Chemie GmbH, Germany), 25 fmol/μl des-Pro2-Bradykinin (Sigma-Aldrich) and 50 fmol/μl Adrenocorticotropic Hormone Fragment 18-39 human (Sigma-Aldrich).

#### Acquisition of Mass Spectra

A MALDI-TOF/TOF MS instrument (4800 Proteomics Analyser Plus; Applied Biosystems, Foster City, USA) was used to acquire peptide mass fingerprints (PMFs) and peptide sequence spectra. Each MALDI plate was calibrated according to the manufacturer's specifications. All PMF spectra were internally calibrated with the spiked internal standards, resulting in an average mass accuracy of 5 ± 10 ppm. Using the individual PMF spectra, up to eight peptides exceeding a signal-to-noise ratio of 100 that passed through a mass exclusion filter were submitted to CID fragmentation. The subsequent fragmentation spectra were calibrated using default calibration.

#### MS-based Protein Homology Identification

PMF and peptide sequence spectra of each sample were processed with the accompanied software suit (GPS Explorer 3.5 - Applied Biosystems) and submitted for protein homology identification by using a local database search engine (Mascot 2.1; Matrix Science, London, UK) (SwissProt, Viridiplantae (Green Plants), 27901 sequences, 27 July 2009 at 11:27:15 GMT)). The maximum number of missed cleavages was set to two. The variable modifications selected for searching includd oxidation of methionine and carbamidomethylation of cystein. A peptide mass tolerance of ± 40 ppm, a fragment mass tolerance of ± 0.4 Da and a peptide charge of + 1 were selected. Only significant hits, as defined by the MASCOT probability analysis (p < 0.05), were accepted.

### 5.5. IEF/SDS-PAGE

#### Protein labelling and experimental design

The proteins were labelled (stroma: 50 μg proteins) with 200 pmol of the CyDyes, according to the manufacturer's protocol (Amersham Biosciences/GE Healthcare). The proteins from wild-type and transgenic plants were labelled with Cy3 and Cy5. A dye swop was included to minimize the effects of dye-specific labelling. An internal standard generated by pooling equal amounts of proteins from each sample was labelled with Cy2. Differentially labelled samples were immediately combined in a 1:1:1 ratio; rehydration buffer (7 M urea, 2 M thiourea, 4% w/v CHAPS, 0.005% bromphenol blue) containing 60 mM DTT and 2% IPG buffer (GE Healthcare) was added to make up the volume to 150 μl. Thereafter, the samples were subjected to protein separation by two-dimensional PAGE.

#### 2-D gel electrophoresis

After overnight rehydratation of the Immobiline dry strips (NL, pH 3-10; GE Healthcare) with Destreak, pooled labelled protein samples were separated on an IPGPhor Unit (GE Healthcare) using the following settings: 1 h 250 V (step), 7 h 1,000 V (gradient), 3 h 8,000 V (step), 3 h 45 min 8,000 V (gradient) for a total of 49.2 kVh (50 μA/strip, 20°C). After IEF, the strips were equilibrated for 15 min in equilibration buffer (50 mM Tris/HCl, pH 8.8, 6 M urea, 30% v/v glycerol, 2% w/v SDS, 0.01% w/v bromphenol blue) containing 0.1% dithriotreitol (DTT) and for 15 min in equilibration buffer containing 4.25% iodoacetamide. After rinsing the strips with water, they were mounted on top of a 12.5% SDS-polyacrylamide gel and overlaid with agarose sealing solution. SDS-PAGE was carried out in an EttanDALT*six *unit (GE Healthcare) (1 h at 0.1 W/gel, followed by 17 h at 1 W/gel).

#### Gel imaging and data analysis

Proteins were visualized by scanning using an Ettan DIGE Imager (GE Healthcare). All gels were scanned at 100 μm (pixel size) resolution. The scanned gels were then directly transferred to the ImageQuant V5.2 software package (GE Healthcare) and saved as gel format. Gel images were cropped using the ImageQuant TL software programme (GE Healthcare) and gel analysis was carried out with the DeCyder 6.5 software (GE Healthcare). Spot detection was performed using the DIA (differential in-gel analysis) module with an estimated number of spots set to 10,000. After matching the gels using three landmarks, DIGE images were further analysed using the DeCyder BVA (biological variation analysis) module. For each transgenic and wild-type line, images from at least three biological repeats were used for the statistical analysis of protein abundance. A student's t-test in combination with correction for the false discovery rate was performed to identify spots of interest (n = 3, p < 0.05). The differently expressed proteins were manually picked after staining the gels using silver staining (described in section 2.4).

### 5.6. Quantitative RT-PCR

Leaf samples (third leaf larger than 5 cm) were taken from 15 separate plants of each of the two transgenic lines and the two wild-types at the same time in the morning and snap frozen in liquid nitrogen before storage at -70°C. RNA isolation, cDNA synthesis, primer design, and real-time PCR were performed, as described by Cortleven *et al. *[32]. Primer pairs of reference genes and genes of interest are listed in Table 3. Statistical analysis was performed using SAS v. 9.1.3. by a student's t-test. The normality and homogeneity of variance were tested using the Shapiro-Wilk and Levene tests [55]. In order to meet the assumptions, datasets were transformed using log transformation.

**Table 3:** Primer sequences of the used reference genes and genes of interest.

Genes	Accession Number	Primer sequence 5'- 3' Primer sequence 3'- 5'	Primer efficiency
**Reference genes**			
*Nuclear-encoded*			
*ACT9*	X69885	CTATTCTCCGCTTTGGACTTGGCA AGGACCTCAGGACAACGGAAACG	95.67%
*αTUB*	AJ421412.1	GATGTTGTGCCAAAGGATGTCA GGCTGATAGTTGATACCACACTTGAAT	93.43%
*SSU*	X02353	AATGGATGGGTTCCTTGTTT GTATGCCTTCTTCGCCTCTC	107.16%

**Genes of interest**			
*Nuclear-encoded*			
*PSBO*	AY220076	CGTGTGCCCTTCCTCTTCA GATCCACCCCGTCCCTTT	114.10%
*PSBP*	X55354.1	CATTGTCGCTATCACCCCTAC ATACGGTTTCCCTCCCACTT	87.18%
*PSBQ*	AB188569.1	CGTCATCGGTCTTGTTGT CAATCTCCTTGGCTGAATCCT	94.26%
*FNR*	Y14032	CACCAATGACAAAGGGGAAG GAATGAACGGAAAGGAGCAA	92.29%
*SODB*	M55909.1	CCCAACGGAGGAGGAGAG GGAGTTTTCACCAAGGCAAG	90.67%

## Abbreviations

ACC-synthase: 1-aminocyclopropane-1-carboxylic acid synthase; ATP: adenosine-5'-triphosphate; BN: blue-native; CaMV 35S: cauliflower mosaic virus 35S promoter; cDNA: complementary DNA; CF1: hydrophylic headpiece ATP synthase; CKX: gene encoding cytokinin oxidase; ClpA: Caseinolytic protease A; FAD: flavine-adenine-dinucleotide; FNR: ferredoxin-NADP^+^-reductase; GTP: guanosine triphosphate,* IPT*: gene encoding adenosine phosphate-isopentenyl transferase; LHC: light-harvesting complex; NADP: nicotinamide-adenine dinucleotidephosphate,*NtACC*: *Nicotiana tabacum *gene encoding β subunit of acetyl-CoA carboxylase; *NtACT9*: *Nicotiana tabacum *gene encoding actin 9; *NtATPC*: *Nicotiana tabacum *gene encoding gamma subunit of ATP synthase; *NtαTUB*: *Nicotiana tabacum *gene encoding alfa-tubulin; *NtFNR*: *Nicotiana tabacum *gene encoding ferredoxin-NADP-reductase; *NtIN1*: *Nicotiana tabacum *gene encoding initiation factor 1; *NtNDHI*: *Nicotiana tabacum *gene encoding NADH dehydrogenase subunit; *NtPETD*: *Nicotiana tabacum *gene encoding subunit IV of cytochrome *b*_*6*_*f*; *NtPSAA*:*Nicotiana tabacum *gene encoding PSI-A; *NtPSAB*: *Nicotiana tabacum *gene encoding PSI-B; *NtPSBC*: *Nicotiana tabacum *gene encoding PSII CP43; *NtPSBD*: *Nicotiana tabacum *gene encoding D1; *NtPSBE*: *Nicotiana tabacum *gene encoding cytochrome b559; *NtPSBO*: *Nicotiana tabacum *gene encoding 33 kDa of oxygen evolving complex; *NtPSBP*: *Nicotiana tabacum *gene encoding 23 kDa of oxygen evolving complex; *NtPSBQ*: *Nicotiana tabacum *gene encoding 17 kDa of oxygen evolving complex; *NtRBCL*: *Nicotiana tabacum *gene encoding large subunit of RubisCO; *NtRBCS*: *Nicotiana tabacum *gene encoding small subunit of RubisCO; *NtRPS3*: *Nicotiana tabacum *gene encoding ribosomal protein S3; *NtSOD*: *Nicotiana tabacum *gene encoding superoxide Fe dismutase; *NtSSU*: *Nicotiana tabacum *gene encoding small subunit of RubisCO; OEC: oxygen evolving complex; PSI: photosystem I; PSII: photosystem II; P*ssu*: promotor small subunit RubisCO; RNA: ribonucleic acid; ROS: reactive oxygen species; Rubisco: ribulose-1,5-bisphosphate Carboxylase/Oxygenase; SOD: superoxide dismutase.

## Competing interests

The authors declare that they have no competing interests.

## Authors' contributions

AC participated in design of the study, performed all the experimental procedures and drafted the manuscript. JPN participated in the identification of the proteins by MALDI/TOF(TOF). RV conceived of the study, participated in its design and helped to draft the manuscript. All authors read and approved the final manuscript.

## Supplementary Material

Additional file 1 **Table S1. Proteins from the second dimension (BN/SDS-PAGE) identified by MALDI-TOF/TOF**. Include additional info from the identified proteins from the second dimension (BN/SDS-PAGE) such as accession number, p-value, protein score, peptide count, sequence coverage, sequence, ion score, observed precursor mass, mass error and identification method. Click here for file

Additional file 2 **Table S2. Proteins from stroma fraction (IEF/SDS-PAGE) identified by MALDI-TOF/TOF**. Include additional info from the identified proteins from the stroma fraction (IEF/SDS-PAGE) such as accession number, p-value, protein score, peptide count, sequence coverage, sequence, ion score, observed precursor mass, mass error and identification method. Click here for file
