# Supplementary material for: Analysis of the photosynthetic apparatus in transgenic tobacco plants with altered endogenous cytokinin content: a proteomic study
Source: Proteome Sci. 2011 Jun 26;9:33. doi: 10.1186/1477-5956-9-33 (PMC3151202; doi:10.1186/1477-5956-9-33)
Supplement: Additional file 1 — Table S1. Proteins from the second dimension (BN/SDS-PAGE) identified by MALDI-TOF/TOF. Include additional info from the identified proteins from the second dimension (BN/SDS-PAGE) such as accession number, p-value, protein score, peptide count, sequence coverage, sequence, ion score, observed precursor mass, mass error and identification method. [file 1477-5956-9-33-S1.DOC]

**Additional file 1:** Proteins identified by MALDI-TOF/TOF of the second dimension (BN-SDS/PAGE).

| **Spot number** | **Protein Name** | **Accession Number (a)** | **p-value (b)** | **Protein Score** | **Peptide Count (c)** | **Sequence coverage (d)** | **Sequence (e)** | **Ion Score** | **Observed Precursor Mass** | **Mass Error (±Da)** | **Iden-tification method (f)** |
| --- | --- | --- | --- | --- | --- | --- | --- | --- | --- | --- | --- |
| 1 | ATP synthase subunit alpha, chloroplastic [*Nicotiana tabacum*] | P00823 | 8.80E-13 | 165 | 10 | 22% | LIESAAPGIISR | 34 | 1226.7102 | -0.0001 | MS/MS |
| 2 | Photosystem II CP47 chlorophyll apoprotein [*Cucumis sativus*] | Q2QD63 | 0.013 | 63 | 6 | 12% | / | / | / | / | MS |
| 3 | Photosystem II CP43 chlorophyll apoprotein [*Ranunculus macranthus*] | Q4FFN5 | 2.20E-06 | 101 | 9 | 18% | / | / | / | / | MS |
| 4 | Oxygen-evolving enhancer protein 1, [chloroplastic *Nicotiana tabacum*] | Q40459 | 8.80E-09 | 125 | 8 | 29% | / | / | / | / | MS |
| 5 | Chlorophyll a-b binding protein 40, chloroplastic [*Nicotiana tabacum*] | P27495 | 8.80E-12 | 155 | 6 | 13% | FGEAVWFK | 55 | 983.4943 | -0.0042 | MS/MS |
|  |  |  |  |  |  |  | ELEVIHCR | 48 | 1055.5255 | -0.0048 |  |
| 6 | Chlorophyll a-b binding protein 13, chloroplastic [*Solanum lycopersicum*] | P27489 | 1.50E-03 | 73 | 2 | 6% | VDFKEPVWFK | 38 | 1294.6841 | 0.0011 | MS/MS |
| 7 | Chlorophyll a-b binding protein 6A, chloroplastic [*Solanum lycopersicum*] | P12360 | 2.80E-07 | 110 | 5 | 12% | YPGGAFDPLGYSK | 50 | 1371.6609 | 0.0029 | MS/MS |
| 8 | Photosystem I reaction center subunit II, chloroplastic [*Nicotiana sylvestris*] | P29302 | 2.80E-06 | 100 | 5 | 26% | / | / | / | / | MS |
| 9 | Photosystem I reaction center subunit II, chloroplastic [*Nicotiana sylvestris*] | P29302 | 1.80E-08 | 122 | 5 | 26% | QGVGQNFR | 30 | 905.4553 | -0.0034 | MS/MS |
|  |  |  |  |  |  |  | VFPNGEVQYLHPK | 29 | 1527.802 | 0.0066 |  |
| 10 | Photosystem I reaction center subunit III, chloroplastic [*Spinacia oleracea*] | P12355 | 1.40E-02 | 63 | 2 | 7% | GFSWPVAAYR | 38 | 1153.579 | 0.0001 | MS/MS |
| 11 | Photosystem II CP43 chlorophyll apoprotein [*Aethionema grandiflora*] | A4QJJ5 | 6.20E-06 | 96 | 8 | 19% | / | / | / | / | MS |
| 12 | Oxygen-evolving enhancer protein 1, chloroplastic [*Nicotiana tabacum*] | Q40459 | 2.20E-06 | 101 | 9 | 31% | / | / | / | / | MS |
| 13 | Ribulose bisphosphate carboxylase large chain (Fragment) [*Nelumbo lutea*] | Q05800 | 9.70E-03 | 65 | 8 | 18% | / | / | / | / | MS |
| 14 | Photosystem I P700 chlorophyll a apoprotein A1 [*Nicotiana tomentosiformis*] | Q33C36 | 5.80E-04 | 77 | 6 | 8% | / | / | / | / | MS |
| 15 | Photosystem II CP43 chlorophyll apoprotein [*Ranunculus macranthus*] | Q4FFN5 | 0.023 | 61 | 8 | 16% | / | / | / | / | MS |
| 16 | Oxygen-evolving enhancer protein 1, chloroplastic [*Nicotiana tabacum*] | Q40459 | 3.40E-04 | 79 | 8 | 28% | / | / | / | / | MS |
| **Spot number** | **Protein Name** | **Accession Number (a)** | **p-value (b)** | **Protein Score** | **Peptide Count (c)** | **Sequence coverage (d)** | **Sequence (e)** | **Ion Score** | **Observed Precursor Mass** | **Mass Error (±Da)** | **Iden-tification method (f)** |
| 17 | Photosystem II D2 protein [*Dioscorea elephantipes*] | A6MMK2 | 8.00E-06 | 95 | 5 | 16% | NILLNEGIR | 31 | 1041.6042 | -0.0009 | MS/MS |
| 18 | Chlorophyll a-b binding protein 40, chloroplastic [*Nicotiana tabacum*] | P27495 | 1.10E-04 | 84 | 5 | 12% | FGEAVWFK | 34 | 983.4957 | -0.0028 | MS/MS |
| 19 | Chlorophyll a-b binding protein 6A, chloroplastic [*Solanum lycopersicum*] | P12360 | 4.40E-08 | 118 | 3 | 9% | YPGGAFDPLGYSK | 49 | 1371.657 | -0.001 | MS/MS |
|  |  |  |  |  |  |  | KYPGGAFDPLGYSK | 36 | 1499.7551 | 0.0022 |  |
| 20 | Photosystem I reaction center subunit II, chloroplastic [*Nicotiana sylvestris*] | P29302 | 1.10E-07 | 114 | 8 | 39% | VFPNGEVQYLHPK | 30 | 1527.7971 | 0.0017 | MS/MS |
| 21 | Photosystem I reaction center subunit II, chloroplastic [*Nicotiana sylvestris*] | P29302 | 3.10E-06 | 100 | 7 | 30% | VFPNGEVQYLHPK | 29 | 1527.7994 | 0.004 | MS/MS |
| 22 | Photosystem I reaction center subunit IV B, chloroplastic [*Nicotiana sylvestris*] | Q41229 | 0.0088 | 65 | 5 | 39% | / | / | / | / | MS |
| 23 | Photosystem I reaction center subunit III, chloroplastic [*Flaveria trinervia*] | P46486 | 3.20E-04 | 79 | 3 | 15% | GFSWPVAAYR | 44 | 1153.5845 | 0.0056 | MS/MS |
| 24 | Cytochrome b559 subunit alpha [*Arabidopsis thaliana*] | P56779 | 3.50E-09 | 129 | 4 | 46% | FDSLEQLDEFSR | 54 | 1485.6914 | 0.0057 | MS/MS |
| 25 | ATP synthase subunit alpha, chloroplastic [*Nicotiana tabacum*] | P00823 | 3.90E-06 | 99 | 10 | 22% | / | / | / | / | MS |
| 26 | Ribulose bisphosphate carboxylase large chain [*Nicotiana sylvestris*] | Q3C1J4 | 1.10E-52 | 564 | 20 | 45% | NHGIHFR | 58 | 880.4619 | 0.0083 | MS/MS |
|  |  |  |  |  |  |  | DTDILAAFR | 69 | 1021.5263 | -0.0049 |  |
|  |  |  |  |  |  |  | DNGLLLHIHR | 71 | 1187.6692 | 0.0048 |  |
|  |  |  |  |  |  |  | TFQGPPHGIQVER | 65 | 1465.7607 | 0.0061 |  |
| 27 | Ribulose bisphosphate carboxylase large chain (Fragment) [*Adoxa moschatellina*] | P28378 | 7.00E-07 | 106 | 12 | 22% | / | / | / | / | MS |
| 28 | Photosystem II CP47 chlorophyll apoprotein [*Barbarea verna*] | A4QKD1 | 4.40E-10 | 138 | 10 | 18% | / | / | / | / | MS |
| 29 | Photosystem II CP43 chlorophyll apoprotein [*Aethionema grandiflora*] | A4QJJ5 | 1.80E-15 | 192 | 9 | 19% | LGANVGSAQGPTGLGK | 46 | 1426.7648 | 0 | MS/MS |
| 30 | Photosystem Q(B) protein [*Leptosira terrestri*] | A6YGB8 | 2.80E-12 | 160 | 7 | 22% | NAHNFPLDLA | 57 | 1111.559 | 0.0059 | MS/MS |
|  |  |  |  |  |  |  | VLNTWADIINR | 38 | 1314.7161 | -0.0003 |  |
| 31 | Photosystem Q(B) protein [*Leptosira terrestris*] | A6YGB8 | 0.048 | 58 | 7 | 22% | / | / | / | / | MS |
| 32 | Cytochrome b559 subunit alpha [*Arabidopsis thaliana*] | P56779 | 1.80E-12 | 162 | 3 | 37% | QGIPLITGR | 38 | 954.5704 | -0.0027 | MS/MS |
| **Spot number** | **Protein Name** | **Accession Number (a)** | **p-value (b)** | **Protein Score** | **Peptide Count (c)** | **Sequence coverage (d)** | **Sequence (e)** | **Ion Score** | **Observed Precursor Mass** | **Mass Error (±Da)** | **Iden-tification method (f)** |
| 33 | Apocytochrome f [*Nicotiana tabacum*] | P06449 | 2.20E-61 | 651 | 16 | 51% | IPYDMQLK | 46 | 1023.5178 | -0.0001 | MS/MS |
|  |  |  |  |  |  |  | YPIYVGGNR | 60 | 1038.5386 | 0.0019 |  |
|  |  |  |  |  |  |  | NILVIGPVPGQK | 74 | 1234.7494 | -0.0024 |  |
|  |  |  |  |  |  |  | GGYEOTOTDASDGR | 80 | 1454.6744 | -0.0014 |  |
|  |  |  |  |  |  |  | SNNTVYNATAAGIVSK | 103 | 1609.8198 | 0.0018 |  |
|  |  |  |  |  |  |  | EKGGYEITITDASDGR | 58 | 1711.8152 | 0.0018 |  |
| 34 | Chlorophyll a-b binding protein 7, chloroplastic [*Nicotiana tabacum*] | P27491 | 8.80E-11 | 145 | 5 | 19% | FGEAVWFK | 66 | 983.4913 | -0.0072 | MS/MS |
| 35 | Cytochrome b6-f complex iron-sulfur subunit 2, chloroplastic [*Nicotiana tabacum*] | Q02585 | 6.50E-06 | 96 | 5 | 28% | VVFVPWVETDFR | 30 | 1493.7817 | 0.003 | MS/MS |
| 36 | Cytochrome b6-f complex subunit 4 [*Agrostis stolonifera*] | A1EA39 | 9.00E-05 | 85 | 3 | 12% | / | / | / | / | MS |
| 37 | ATP synthase subunit b, chloroplastic [*Nicotiana tabacum*] | P06290 | 1.40E-13 | 173 | 11 | 44% | GVLSDLLDNR | 29 | 1101.5863 | -0.0035 | MS/MS |
| 38 | Ferredoxin--NADP reductase, leaf-type isozyme, chloroplastic [*Nicotiana tabacum*] | O04977 | 0.015 | 63 | 6 | 18% | / | / | / | / | MS |
| 39 | Fructose-bisphosphate aldolase, chloroplastic [*Spinacia oleracea*] | P16096 | 8.80E-11 | 145 | 6 | 13% | SAAYYQQGAR | 38 | 1114.5275 | -0.0001 | MS/MS |
|  |  |  |  |  |  |  | LASIGLENTEANR | 54 | 1387.7217 | 0.0042 |  |
| 40 | Photosystem II 22 kDa protein, chloroplastic [*Nicotiana tabacum*] | Q9SMB4 | 5.60E-10 | 137 | 5 | 17% | ANELFVGR | 42 | 905.4797 | -0.0042 | MS/MS |
| 41 | Apocytochrome f [*Nicotiana tabacum*] | P06449 | 4.40E-61 | 198 | 11 | 40% | NILVIGPVPGQK | 39 | 1234.7452 | -0.0066 | MS/MS |
| 42 | Photosystem II 22 kDa protein, chloroplastic [*Nicotiana tabacum*] | Q9SMB4 | 3.20E-06 | 99 | 5 | 17% | ANELFVGR | 35 | 905.4807 | -0.0032 | MS/MS |
| 43 | Photosystem II CP43 chlorophyll apoprotein [*Aethionema grandiflora*] | A4QJJ5 | 0.02 | 62 | 8 | 19% | / | / | / | / | MS |
| 44 | ATP synthase subunit beta, chloroplastic [*Nicotiana plumbaginifolia*] | P69370 | 2.20E-11 | 151 | 13 | 36% | / | / | / | / | MS |
| 45 | ATP synthase subunit alpha, chloroplastic [*Nicotiana tabacum*] | P00823 | 2.20E-14 | 181 | 11 | 25% | LIESAAPGIISR | 29 | 1226.709 | -0.0013 | MS/MS |
| 46 | Chlorophyll a-b binding protein CP26, chloroplastic [*Arabidopsis thaliana*] | Q9XF89 | 3.10E-02 | 74 | 5 | 21% | / | / | / | / | MS |
| 47 | Ferredoxin--NADP reductase, leaf-type isozyme, chloroplastic [*Nicotiana tabacum*] | O04977 | 5.60E-08 | 117 | 11 | 30% | / | / | / | / | MS |
| **Spot number** | **Protein Name** | **Accession Number (a)** | **p-value (b)** | **Protein Score** | **Peptide Count (c)** | **Sequence coverage (d)** | **Sequence (e)** | **Ion Score** | **Observed Precursor Mass** | **Mass Error (±Da)** | **Iden-tification method (f)** |
| 48 | ATP synthase subunit alpha, chloroplastic [*Nicotiana tabacum*] | P00823 | 1.30E-04 | 83 | 10 | 22% | / | / | / | / | MS |
| 49 | ATP synthase subunit alpha, chloroplastic [*Nicotiana tabacum*] | P00823 | 1.10E-09 | 134 | 12 | 24% | / | / | / | / | MS |
| 50 | ATP synthase gamma chain, chloroplastic [*Nicotiana tabacum*] | P29790 | 0.068 | 66 | 7 | 19% | / | / | / | / | MS |
| 51 | ATP synthase subunit beta, chloroplastic [*Nicotiana tabacum*] | P00826 | 6.80E-06 | 77 | 10 | 32% | / | / | / | / | MS |
| 52 | Oxygen-evolving enhancer protein 1, chloroplastic [*Nicotiana tabacum*] | Q40459 | 0.0006 | 84 | 8 | 29% | / | / | / | / | MS |

1. Accession number based on SwissProt
2. Measure of certainty for each identified protein
3. Number of peptides that match the sequence of the identified protein
4. Total percentage of sequence coverage of the found peptides
5. Sequence of the peptides used to identify the protein in MS/MS
6. Identification method: either MS of MS/MS
