# Supplementary material for: Analysis of the photosynthetic apparatus in transgenic tobacco plants with altered endogenous cytokinin content: a proteomic study
Source: Proteome Sci. 2011 Jun 26;9:33. doi: 10.1186/1477-5956-9-33 (PMC3151202; doi:10.1186/1477-5956-9-33)
Supplement: Additional file 2 — Table S2. Proteins from stroma fraction (IEF/SDS-PAGE) identified by MALDI-TOF/TOF. Include additional info from the identified proteins from the stroma fraction (IEF/SDS-PAGE) such as accession number, p-value, protein score, peptide count, sequence coverage, sequence, ion score, observed precursor mass, mass error and identification method. [file 1477-5956-9-33-S2.DOC]

<Table 2> Proteins identified by MALDI-TOF/TOF from the second dimension BN/SDS-PAGE. Additional information about the identified proteins can be found in Additional file 1.

| **Spot number** | **Protein Name** | **SwissProt Accession number** | **MW** | **pI** |
| --- | --- | --- | --- | --- |
| 1 | ATP synthase subunit alpha, chloroplastic [*Nicotiana tabacum*] | P00823 | 55477,1 | 5,14 |
| 2 | Photosystem II CP47 chlorophyll apoprotein [*Cucumis sativus*] | Q2QD63 | 55982,37 | 6,28 |
| 3 | Photosystem II CP43 chlorophyll apoprotein [*Ranunculus macranthus*] | Q4FFN5 | 52017,79 | 6,68 |
| 4 | Oxygen-evolving enhancer protein 1, [chloroplastic *Nicotiana tabacum*] | Q40459 | 35377,09 | 5,89 |
| 5 | Chlorophyll a-b binding protein 40, chloroplastic [*Nicotiana tabacum*] | P27495 | 28450,22 | 5,48 |
| 6 | Chlorophyll a-b binding protein 13, chloroplastic [*Solanum lycopersicum*] | P27489 | 28661,44 | 5,09 |
| 7 | Chlorophyll a-b binding protein 6A, chloroplastic [*Solanum lycopersicum*] | P12360 | 26785,52 | 5,82 |
| 8 | Photosystem I reaction center subunit II, chloroplastic [*Nicotiana sylvestris*] | P29302 | 22466,7 | 9,78 |
| 9 | Photosystem I reaction center subunit II, chloroplastic [*Nicotiana sylvestris*] | P29302 | 22466,7 | 9,78 |
| 10 | Photosystem I reaction center subunit III, chloroplastic [*Spinacia oleracea*] | P12355 | 25567,52 | 9,4 |
| 11 | Photosystem II CP43 chlorophyll apoprotein [*Aethionema grandiflora*] | A4QJJ5 | 52042,77 | 6,71 |
| 12 | Oxygen-evolving enhancer protein 1, chloroplastic [*Nicotiana tabacum*] | Q40459 | 35377,09 | 5,89 |
| 13 | Ribulose bisphosphate carboxylase large chain (Fragment) [*Nelumbo lutea*] | Q05800 | 44231,36 | 6,4 |
| 14 | Photosystem I P700 chlorophyll a apoprotein A1 [*Nicotiana tomentosiformis*] | Q33C36 | 83206,56 | 6,67 |
| 15 | Photosystem II CP43 chlorophyll apoprotein [*Ranunculus macranthus*] | Q4FFN5 | 52017,79 | 6,68 |
| 16 | Oxygen-evolving enhancer protein 1, chloroplastic [*Nicotiana tabacum*] | Q40459 | 35377,09 | 5,89 |
| 17 | Photosystem II D2 protein [*Dioscorea elephantipes*] | A6MMK2 | 39765,89 | 5,34 |
| 18 | Chlorophyll a-b binding protein 40, chloroplastic [*Nicotiana tabacum*] | P27495 | 28450,22 | 5,48 |
| 19 | Chlorophyll a-b binding protein 6A, chloroplastic [*Solanum lycopersicum*] | P12360 | 26785,52 | 5,82 |
| 20 | Photosystem I reaction center subunit II, chloroplastic [*Nicotiana sylvestris*] | P29302 | 22466,7 | 9,78 |
| 21 | Photosystem I reaction center subunit II, chloroplastic [*Nicotiana sylvestris*] | P29302 | 22466,7 | 9,78 |
| 22 | Photosystem I reaction center subunit IV B, chloroplastic [*Nicotiana sylvestris*] | Q41229 | 15214,74 | 9,74 |
| 23 | Photosystem I reaction center subunit III, chloroplastic [*Flaveria trinervia*] | P46486 | 25366,48 | 9,35 |
| 24 | Cytochrome b559 subunit alpha [*Arabidopsis thaliana*] | P56779 | 9380,7 | 4,83 |
| 25 | ATP synthase subunit alpha, chloroplastic [*Nicotiana tabacum*] | P00823 | 55477,1 | 5,14 |
| 26 | Ribulose bisphosphate carboxylase large chain [*Nicotiana sylvestris*] | Q3C1J4 | 53377,98 | 6,41 |
| 27 | Ribulose bisphosphate carboxylase large chain (Fragment) [*Adoxa moschatellina*] | P28378 | 52159,14 | 6,13 |
| **Spot number** | **Protein Name** | **SwissProt Accession number** | **MW** | **pI** |
| 28 | Photosystem II CP47 chlorophyll apoprotein [*Barbarea verna*] | A4QKD1 | 56204,47 | 6,4 |
| 29 | Photosystem II CP43 chlorophyll apoprotein [*Aethionema grandiflora*] | A4QJJ5 | 52042,77 | 6,71 |
| 30 | Photosystem Q(B) protein [*Leptosira terrestri*] | A6YGB8 | 38353,08 | 5,52 |
| 31 | Photosystem Q(B) protein [*Leptosira terrestris*] | A6YGB8 | 38353,08 | 5,52 |
| 32 | Cytochrome b559 subunit alpha [*Arabidopsis thaliana*] | P56779 | 9380,7 | 4,83 |
| 33 | Apocytochrome f [*Nicotiana tabacum*] | P06449 | 35337,77 | 9,12 |
| 34 | Chlorophyll a-b binding protein 7, chloroplastic [*Nicotiana tabacum*] | P27491 | 28488,24 | 5,72 |
| 35 | Cytochrome b6-f complex iron-sulfur subunit 2, chloroplastic [*Nicotiana tabacum*] | Q02585 | 24491,28 | 8,15 |
| 36 | Cytochrome b6-f complex subunit 4 [*Agrostis stolonifera*] | A1EA39 | 17534,51 | 6,56 |
| 37 | ATP synthase subunit b, chloroplastic [*Nicotiana tabacum*] | P06290 | 20917,96 | 8,76 |
| 38 | Ferredoxin--NADP reductase, leaf-type isozyme, chloroplastic [*Nicotiana tabacum*] | O04977 | 40704,59 | 8,37 |
| 39 | Fructose-bisphosphate aldolase, chloroplastic [*Spinacia oleracea*] | P16096 | 42726,8 | 6,85 |
| 40 | Photosystem II 22 kDa protein, chloroplastic [*Nicotiana tabacum*] | Q9SMB4 | 29068,73 | 6,04 |
| 41 | Apocytochrome f [*Nicotiana tabacum*] | P06449 | 35337,77 | 9,12 |
| 42 | Photosystem II 22 kDa protein, chloroplastic [*Nicotiana tabacum*] | Q9SMB4 | 29068,73 | 6,04 |
| 43 | Photosystem II CP43 chlorophyll apoprotein [*Aethionema grandiflora*] | A4QJJ5 | 52042,77 | 6,71 |
| 44 | ATP synthase subunit beta, chloroplastic [*Nicotiana plumbaginifolia*] | P69370 | 53548,81 | 5,09 |
| 45 | ATP synthase subunit alpha, chloroplastic [*Nicotiana tabacum*] | P00823 | 55477,1 | 5,14 |
| 46 | Chlorophyll a-b binding protein CP26, chloroplastic [*Arabidopsis thaliana*] | Q9XF89 | 30194,7 | 6 |
| 47 | Ferredoxin--NADP reductase, leaf-type isozyme, chloroplastic [*Nicotiana tabacum*] | O04977 | 40704,59 | 8,37 |
| 48 | ATP synthase subunit alpha, chloroplastic [*Nicotiana tabacum*] | P00823 | 55477,1 | 5,14 |
| 49 | ATP synthase subunit alpha, chloroplastic [*Nicotiana tabacum*] | P00823 | 55477,1 | 5,14 |
| 50 | ATP synthase gamma chain, chloroplastic [*Nicotiana tabacum*] | P29790 | 41705,96 | 8,16 |
| 51 | ATP synthase subunit beta, chloroplastic [*Nicotiana tabacum*] | P00826 | 53577,78 | 5 |
